# Peptidoglycan-associated lipoprotein of *Aggregatibacter actinomycetemcomitans* induces apoptosis and production of proinflammatory cytokines via TLR2 in murine macrophages RAW 264.7 *in vitro*

**DOI:** 10.1080/20002297.2018.1442079

**Published:** 2018-03-06

**Authors:** Riikka Ihalin, Kjell Eneslätt, Sirkka Asikainen

**Affiliations:** a Department of Odontology, Oral Microbiology, Umeå University, Umeå, Sweden; b Department of Biochemistry, University of Turku, Turku, Finland

**Keywords:** *Aggregatibacter actinomycetemcomitans*, apoptosis, mouse macrophages, pro-inflammatory cytokines, peptidoglycan-associated lipoprotein

## Abstract

Peptidoglycan-associated lipoprotein (PAL) is a conserved pro-inflammatory outer membrane lipoprotein in Gram-negative bacteria. Compared to systemic pathogens, little is known about the virulence properties of PAL in *Aggregatibacter actinomycetemcomitans* (AaPAL). The aims of this study were to investigate the cytolethality of AaPAL and its ability to induce pro-inflammatory cytokine production in macrophages. Mouse macrophages were stimulated with AaPAL, and the production of IL-1β, IL-6, TNF-α, and MCP-1 was measured after 6, 24, and 48 h. To investigate which receptor AaPAL employs for its interaction with macrophages, anti-toll-like receptor (TLR)2 and anti-TLR4 antibodies were used to block respective TLRs on macrophages. Metabolic activity and apoptosis of the macrophages were investigated after stimulation with AaPAL. AaPAL induced the production of MCP-1, TNF-α, IL-6, and IL-1β from mouse macrophages in order of decreasing abundance. The pre-treatment of macrophages with an anti-TLR2 antibody significantly diminished cytokine production. Under AaPAL stimulation, the metabolic activity of macrophages decreased in a dose- and time-dependent manner. Furthermore, AaPAL induced apoptosis in 56% of macrophages after 48 h of incubation. Our data suggest that AaPAL can kill macrophages by apoptosis. The results also emphasize the role of AaPAL as a potent pro-inflammatory agent in *A. actinomycetemcomitans*-associated infections.

## Introduction

Periodontitis is a multifactorial inflammatory disease which destroys tooth-supporting tissues; however, it may also correlate with the development of systemic disorders such as cardiovascular diseases when bacteria and their inflammatory components are released to the circulation from inflamed periodontal pockets [1]. Gram-negative species are over-represented in periodontitis compared to healthy periodontium microbiota. Thus, the spread of lipopolysaccharide (LPS) from periodontal pockets eliciting systemic inflammatory host responses has been suggested to accelerate atherogenesis [2]. *Aggregatibacter actinomycetemcomitans* is a Gram-negative oral bacterium which is a major pathogen in aggressive periodontitis, especially in populations with North-West African descent when colonized by the highly leukotoxic serotype b JP2-clone of the bacterium. Thus, it has extensively been used as a model species in studies investigating the etiopathogenesis of this disease. Although *A. actinomycetemcomitans* is an oral bacterium, it can cause severe non-oral infections such as endocarditis and abscesses in brains and lungs on rare occasions [3–5]. LPS is the best-known pro-inflammatory outer membrane component in *A. actinomycetemcomitans* [6–8].

The role of LPS in infections caused by Gram-negative bacteria has been widely studied (for review, see ref [9].). However, current interest concerning Gram-negative infections is increasingly focused on bacterial outer membrane proteins (OMPs), especially lipoproteins [10]. In fact, recent studies have suggested that bacterial lipoproteins are major players in inflammatory reactions caused by pathogenic species, such as *Shigella flexneri, Brucella abortus*, and *Salmonella enterica* [11–13]. Regarding *A. actinomycetemcomitans*, 23 different lipoproteins are detectable among the proteins secreted by *A. actinomyctemcomitans* biofilm [14], but their bioactivity is poorly understood [15–17]. More knowledge is available for other AaOMPs (e.g. a 100-kDa OMP), which promotes adherence and invasion to human cells, and induction of IL-1b and TNF-a production by mouse macrophages [18]. Furthermore, a 29-kDa AaOMP is involved in bacterial internalization in gingival epithelial cells [17], and the 12-kDa OapB most likely inhibits the host defence enzyme lysozyme [14,19].

Peptidoglycan-associated lipoprotein (PAL) is a conserved outer membrane lipoprotein of Gram-negative bacteria. It is an important member of the TOL-PAL family which is involved in maintaining the cell wall organization [20], but it also functions as a pro-inflammatory agent in Gram-negative infections. For example, *Escherichia coli* PAL is released into the bloodstream and has a pathogenic role in Gram-negative sepsis in a mouse model [21], and a form of *E. coli* PAL which is released into human serum *in vitro* is able to induce pro-inflammatory cytokine production by mouse macrophages [22]. Furthermore, a *Hemophilus ducrey* PAL-deficient mutant strain was less virulent than its parental strain in a human infection model [23], and *B. abortus* PAL is one of the lipoproteins which causes production of pro-inflammatory cytokines in brucellosis [12]. *Klebsiella pneumoniae* PAL protects bacterial cells from serum killing, as well as phagocytosis, and is an important virulence factor in a mouse infection model [24]. In addition, strongly immunogenic PAL of *Burkholderia cenocepacia* [25] facilitates attachment of this opportunistic pathogen to the lung epithelial cells and stimulates IL-8 secretion in cystic fibrosis [26].

We found that a 17-kDa AaOMP which was identified as the PAL of *A. actinomycetemcomitans* (AaPAL) [16] is released by planktonic and biofilm cultures in a soluble form [14,27], which suggests that this molecule can readily disseminate from periodontal pockets into the blood circulation. A significant antibody response to AaPAL in sera of *A. actinomycetemcomitans*-positive periodontitis patients, but not in the sera of healthy subjects [16], gives evidence of the systemic effect of AaPAL *in vivo*. In this study, the immunoaffinity chromatography-purified AaPAL [28] serves to further investigate its biological activity. Here, we report the ability of AaPAL to induce apoptosis in addition to pro-inflammatory cytokine production by mouse macrophages in a TLR2-dependent manner.

## Methods

### Purification of AaPAL

AaPAL was purified as described in detail by Ihalin et al. [28]. Briefly, the *A. actinomycetemcomitans* strain D7SS (serotype a), a gift from Dr. Casey Chen (University of Southern California, Los Angeles, CA), was cultured in yeast extract and glucose-supplemented Trypticase soy broth to OD_600_ _nm_ = 0.6. In this study, the same *A. actinomycetemcomitans* strain was used from which AaPAL was originally identified [16] and characterized [28]. The purification protocol consisted of two steps: 1) the crude preparation [29,30] of AaPAL, based on the insolubility of the peptidoglycan, and 2) the differences in the dissociation conditions of different peptidoglycan-binding proteins, followed by the final purification using immunoaffinity chromatography with AaPAL anti-peptide antibodies.

### LPS purification

LPS to be used in macrophage stimulations was purified from *A. actinomycetemcomitans* strain D7SS (serotype a) using a modification of the method by al-Hendy et al. [31]. Briefly, bacterial cells of plate culture of *A. actinomycetemcomitans* were washed once with PBS and once with TEA-buffer (2 mM EDTA, 40 mM Tris-acetate, pH 8.5). The bacterial pellet was suspended in TEA-buffer, 2 X volume of alkaline solution (3% SDS, 0.6% Trizma-base, 128 mM NaOH) was added, and the suspension was incubated at 60°C for 70 min. An equal volume of pre-heated phenol-chloroform (1:1 vol/vol) was mixed with the suspension, which was then incubated at 60°C for 15 min. The mixture was centrifuged (16,000 g, 10 min) and the upper phase was digested with proteinase K (final concentration 0.19 mg/mL) at 60°C for 60 min. After digestion, 1/10 volume of sodium acetate solution (3 M, pH 5.2) was added, vortexed, and precipitated with 2 X volume of ice-cold 99.5% ethanol at −80°C for 20 min. The precipitate was separated by centrifugation (16,000 g, 4°C, 10 min) and suspended in Tris-buffered sodium acetate (100 mM sodium acetate, 50 mM Tris, pH 8.0) by vortexing. LPS was precipitated with ice-cold ethanol and separated by centrifugation as described above, after which the LPS pellet was dried using a SpeedVac concentrator (Savant) for 5 min. The purified LPS was suspended in H_2_O and stored at −80°C until used.

### Chemical assays

The Lowry [32] method was used to determine protein concentrations, and the LPS concentration was estimated using the Limulus assay (Associates of Cape Cod, Inc., East Falmouth, MA) for endotoxins of Gram-negative bacteria.

### Cell cultures

The mouse macrophage ATCC cell line RAW 264.7 was used in stimulation experiments. The cells were cultured in Dulbecco’s modified eagle’s medium (D-MEM) supplemented with 10% foetal calf serum (FCS), 4 mM L-glutamine, 100 U/mL penicillin and 100 μg/mL streptomycin (Gibco, Fisher Scientific), all cell culture grade. For subculturing, the cells were detached using a cell scraper, washed once with the above-described medium, counted using trypan blue staining, and transferred to a new cell culture flask to a final cell density of 50,000 cells/mL. The cells were cultured in 5% CO_2_ at 37°C. The growth medium was changed every 2–4 days, and the next passage was done when the cells were cultured to approximately 80% confluency. Passages 4–8 were used in all experiments.

For the stimulation experiments, the confluent cells were harvested with a cell scraper, washed twice with the medium, and counted using trypan blue staining. The detached cells were centrifuged (233 g, 10 min), suspended in growth medium, and plated onto a 96-well plate at a plating density of 1.65×10^5^ per well. The cells were allowed to adhere to the plastic overnight in 5% CO_2_ at 37°C and then washed once with 200 mL medium/well to remove the non-adherent cells.

### Effect of AaPAL on the metabolic activity of macrophages

The cells were cultured as described above and stimulated with 250 μL of medium containing 0.5, 1.0, 1.2, 1.4, 1.6, 1.8, or 2.0 μg/mL purified AaPAL. In addition, controls with 0.01 ng/mL of LPS were added to every experiment. As LPS in known to be a powerful immune stimulator, and our AaPAL preparation contained LPS as impurity [28], we added the above-mentioned LPS control to show the stimulatory effect of the LPS concentration which was in the highest amount of AaPAL tested. Supplemented D-MEM was used as a negative control. Separate plates were made for measuring the cell metabolic activity after 6, 24, and 48 h of incubation in 5% CO_2_ at 37°C. After incubation, the medium was removed and 200 µL of fresh medium was added together with 22 µL of AlamarBlue™ (bioSource International) color suspension. AlamarBlue™ is reduced and changes its color from blue to red when taken into living cells with a naturally reducing interior environment. After incubation with AlamarBlue™ in 5% CO_2_ at 37°C for 4 h, the absorbance was read at 570 nm and 600 nm, and the amount of reduced AlamarBlue™ was counted as instructed by the supplier using an appropriate correction factor. The final results were calculated as percentage values from the negative medium control. The simulations were repeated three times, from which mean values and standard deviations were calculated.

### Cytokine production of macrophages stimulated with AaPAL

The cells were cultured as described above and stimulated with 250 μL of medium containing 0.5, 1.0, or 2.0 μg/mL purified AaPAL. In addition, control with 0.01 ng/mL LPS was added in every experiment to show the stimulatory effect of LPS in the presence of the highest amount of AaPAL. Supplemented D-MEM was used as a negative control. Separate plates were made for measuring the production of interleukin (IL)-1b, IL-6, tumor necrosis factor (TNF)-a, and monocyte chemotactic protein (MCP)-1 after 6, 24, and 48 h of incubation in 5% CO_2_ at 37°C. After the above-mentioned incubation times, the medium were removed, centrifuged (1,000 g, 5 min), and stored at −70°C. Determination of the cytokine amounts was measured by ELISA-based kits (R&D Systems).

### Role of TLR2 and TLR4 in AaPAL-induced cytokine production

The cells cultured as described above were first pre-treated with 50 µL of supplemented D-MEM containing 4 µg of anti-mouse TLR2 antibody, anti-mouse TLR4 antibody, or isotype controls (eBiosciences), in 5% CO_2_ at 37°C for 2 h. Each series also contained 50 µL of D-MEM without any added antibody to show the basal stimulation effect of the studied substance. After incubation, the medium was replaced by 250 µL of fresh D-MEM supplemented with either 1.5 µg/mL AaPAL preparation, 1 µg/mL synthetic bacterial lipopeptide Pam_3_CSK_4_ (EMC microcollections GmbH), 1.5 µg/mL bovine serum albumin (Fraction V, Roche Diagnostics Corporation), or no additional component. BSA was added to the experiments, because our AaPAL preparation contained small amounts of BSA as a contaminant [28]. The macrophages were stimulated in 5% CO_2_ at 37°C for 24 h, after which the medium samples were collected and stored and the amount of produced cytokines analysed as described above.

### Induction of apoptosis by AaPAL

The cells were stimulated with either 0.5 or 2.0 µg/mL AaPAL, 1 µg/mL synthetic bacterial lipopeptide Pam_3_CSK_4_, or 2.0 μg/mL LPS. Pure D-MEM was used as negative control to detect the baseline apoptosis. After incubation for 48 h in 5% CO_2_ at 37°C, the plates were centrifuged at 1,000 g, the medium was removed, and the cells were washed twice with PBS at room temperature. Labeling of the cells was carried out with the TiterTACS kit (R&D Systems) according to the manufacturer’s instructions. The reaction was stopped with 2 M HCl, and absorbance at 450 nm was measured using a plate reader. Nuclease-treated cells were used as a positive control and regarded as 100% apoptotic cells. TdT enzyme was omitted from some samples to see the non-specific binding of the streptavidin-HRP and regarded as the negative control with 0% apoptotic cells.

### Statistical analysis

The statistical significance of the differences between the AaPAL-treated and non-treated cells, as well as cells treated with anti-TLR2 antibody, anti-TLR4, or non-antibody, were evaluated using the Mann-Whitney U-test. The time dependency of IL-6 and MCP-1 production was evaluated using linear regression analysis. A *p*-value of less than 0.05 was considered statistically significant.

## Results

### Effect of AaPAL on the metabolic activity of macrophages

**Figure 1.**
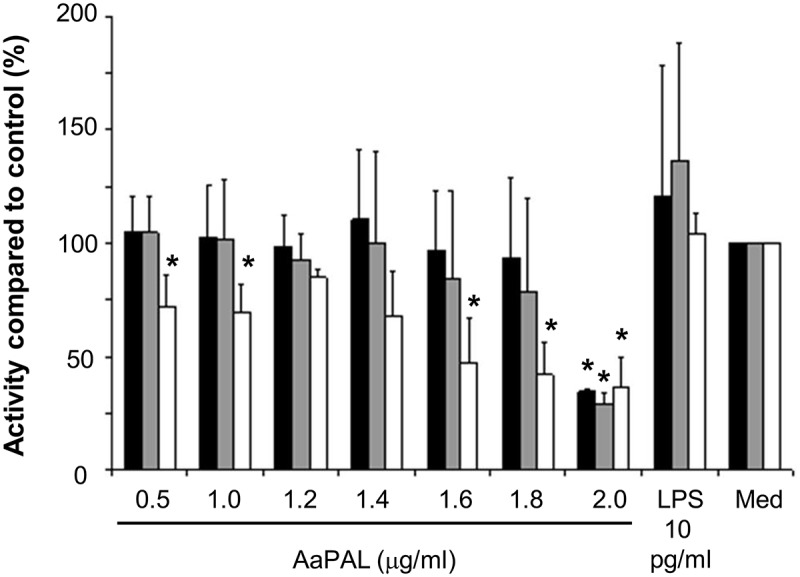
AaPAL induced decrease of metabolic activity in the mouse macrophage cell line RAW 264.7. Cells were incubated with different concentrations of AaPAL for 6 (black), 24 (grey), and 48 h (white). LPS (10 pg/mL) was added to show the stimulatory effect of LPS present in the highest concentration of AaPAL preparation. Supernatant was collected, and the metabolic activity of the cells was determined with AlamarBlue™ (bioSource International). The graph shows means and SD from three separate experiments. * denotes a statistically significant difference (*p* < 0.05; Mann-Whitney U-test) between the tested substance and medium control.

AaPAL decreased the metabolic activity of macrophages in a dose- and time-dependent manner (Figure 1). The highest concentration (2.0 µg/mL) of AaPAL decreased the metabolic activity to 40% of the medium control already after a 6-h incubation; whereas, the same incubation time with lower concentrations had no effect. *A. actinomycetemcomitans* LPS (0.01 ng/mL) increased the metabolic activity of the cells after 6 and 24 h of incubation.

### Cytokine production of macrophages stimulated with AaPAL

**Figure 2.**
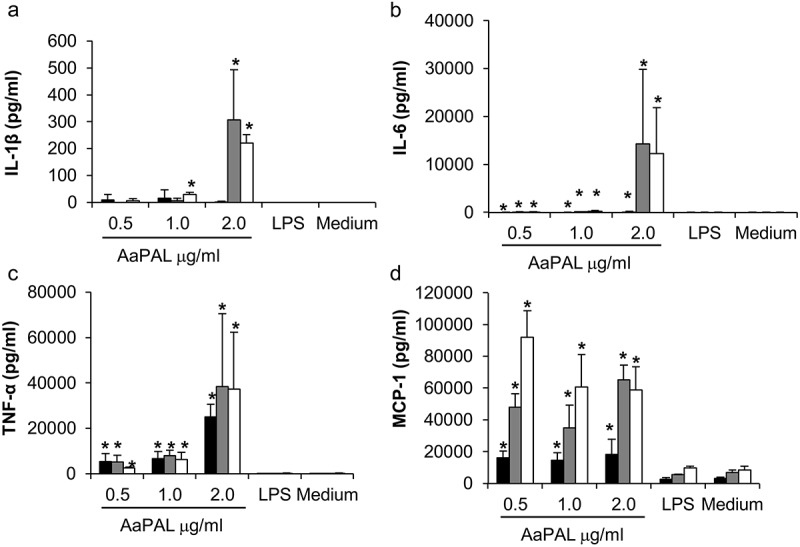
AaPAL induced production of (a) IL-1β, (b) IL-6, (c) TNF-α, and (d) MCP-1 from the mouse macrophage cell line RAW 264.7 as related to measured metabolic activity of the cells (Figure 1). Cells were incubated with different concentrations of AaPAL for 6 (black), 24 (grey), or 48 h (white). LPS (10 pg/mL) was added to show the stimulatory effect of LPS present in the highest concentration of AaPAL preparation. Supernatant was collected, and the produced cytokine amounts were determined using ELISA kits (R&D Systems). The graph shows means and SD from three separate experiments as related to the measured metabolic activities of the cells (Figure 1). * denotes a statistically significant difference (*p* < 0.05; Mann-Whitney U-test) between the tested substance and medium control.

IL-1β, IL-6, and TNF-a production by macrophages was stimulated dose-dependently by AaPAL (Figure 2) when related to the metabolic activity (Figure 1) of the cells. LPS (0.01 ng/mL) did not stimulate production of any studied cytokines more than compared to the culture medium alone (Figure 2).

### Kinetics of cytokine production

**Figure 3.**
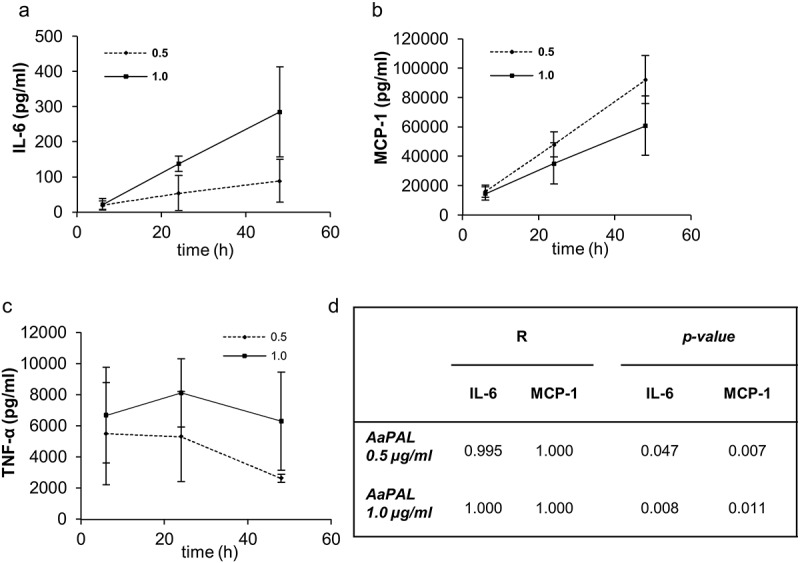
Kinetics of AaPAL induced (a) IL-6, (b) MCP-1, and (c) TNF-α production from the mouse macrophage cell line RAW 264.7. The measured cytokine concentrations as related to the metabolic activity of the cells (Figure 2) were plotted versus time. The graphs show means and SD from three separate experiments as related to metabolic activities. (d) The linear regression analysis and *p*-value for R were calculated for the time dependency of IL-6 and MCP-1 production.

The production of IL-6 and MCP-1, when related to the metabolic activity of the cells, was linearly time-dependent when macrophages were stimulated with 0.5 and 1.0 µg/mL AaPAL (Figure 3). The production of TNF-α did not show a similar time dependency (Figure 3).

### Role of TLR2 and TLR4 in AaPAL-induced cytokine production

**Figure 4.**
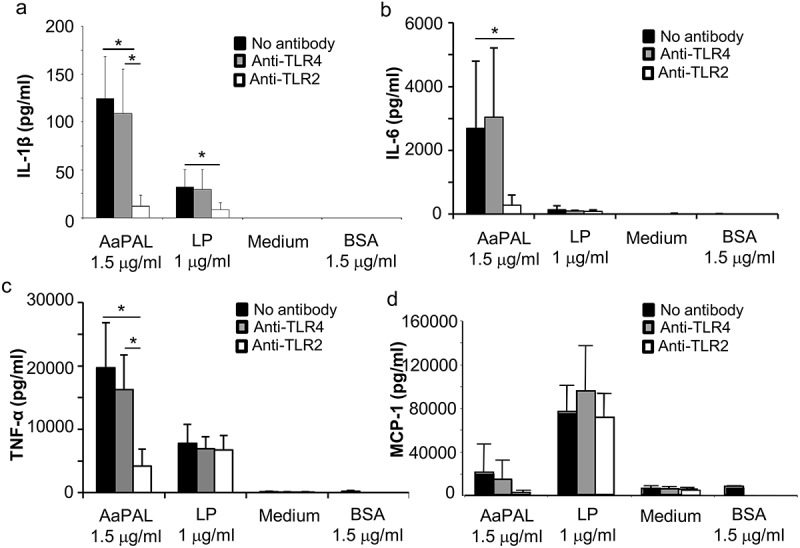
TLR2 dependency of AaPAL-induced cytokine production from the mouse macrophage cell line RAW 264.7. Before incubation with AaPAL for 24 h, the cells were treated with 4 µg of anti-mouse TLR2 (white), anti-mouse (grey), isotype controls (not shown), or no antibody (black) for 2 h. LP denotes synthetic bacterial lipopeptide Pam_3_CSK_4_ (EMC Microcollections GmbH). Bovine serum albumin (BSA) was added to the experiments because the AaPAL preparation contained small amounts of BSA as a contaminant [28]. Supernatant was collected, and the produced amounts of the cytokines (a) IL-1β, (b) IL-6, (c) TNF-α, and (d) MCP-1 were determined using ELISA kits (R&D Systems). The graph shows means and SD from three separate experiments. * denotes a statistically significant difference (*p* < 0.05; Mann-Whitney U-test) between bars connected by the line.

AaPAL induced the production of IL-1b, IL-6, TNF-a, and MCP-1 in a TLR2-dependent manner, because only the pre-incubation of macrophages with anti-TLR2 antibodies significantly decreased the production of cytokines (Figure 4). A similar effect was observed with synthetic lipopeptide Pam_3_CSK_4_ only when the production of IL-1β was measured; however, the anti-TLR2 antibodies did not have any effect on the lipopeptide-induced IL-6, TNF-α or MCP-1 production.

### Induction of apoptosis by AaPAL

AaPAL had a pro-apoptotic effect on the macrophages after a 48-h incubation (Table 1). Incubation with 2.0 μg/mL of AaPAL induced cell death in 56% of the macrophages, although the synthetic lipopeptide Pam_3_CSK_4_ only had a minor apoptosis-inducing effect. Incubation with 2.0 μg/mL of LPS did not show any pro-apoptotic effect.

**Table 1.** AaPAL induced apoptosis of the mouse macrophage cell line RAW 264.7.

	Apoptosis (%)		
	Mean	SD	p-value
			
Medium	23	21	
			
AaPAL 0.5 µg/mL	32	37	0.471
			
AaPAL 2.0 µg/mL	56	33	0.045
			
LP 1.0 µg/mL	30	24	0.044
			
LPS 2.0 µg/mL	20	19	0.628
			

Cells were stimulated with different concentrations of AaPAL for 48 h. The apoptosis was estimated by measuring DNA fragmentation using the TiterTACS kit (R&D systems). LP denotes synthetic bacterial lipopeptide Pam_3_CSK_4_ (EMC Microcollections GmbH). Nuclease-treated cells were used as a positive control and regarded as 100% apoptotic cells. The table shows means and SD from three separate experiments. The statistical significance of the difference between the treated and non-treated (medium) cells was evaluated using the Mann-Whitney U-test. *p*-values <0.05 were considered statistically significant.

## Discussion

We demonstrate in the present study that AaPAL, a structural OMP and lipoprotein of the periodontal pathogen *A. actinomycetemcomitans*, possesses apoptotic activity against mouse macrophages, and using the same cell line, we further showed that AaPAL is a potent inducer of pro-inflammatory cytokines via TLR2. The mouse macrophage RAW 264.7 cell line was selected for the study, because LPS from various *A. actinomycetemcomitans* strains was shown to induce the inflammatory cytokine production and foam cell formation from the same murine cells in our earlier study [33]. To confirm similar effects of AaPAL in humans, human macrophages need to be studied, because it is known that ligands recognized by mouse TLRs may not sometimes stimulate human TLRs.

Our results based on DNA fragmentation of the AaPAL-treated RAW 264.7 cells indicate apoptosis. This was a new finding, because no previous data were found regarding the apoptotic activity of the PAL of any other bacteria. However, studies have shown contradictory results for the apoptotic ability of other bacterial lipoproteins. Whereas most studies suggest that lipoproteins do induce apoptosis [34–36], others report that lipoproteins in fact have anti-apoptotic properties [37]. The observed anti-apoptotic effects could be due to the lipoprotein-stimulated production of TNF-α, as indicated by the finding that LPS-induced long-term survival of macrophages depended on TNF-α autocrine signalling [38]. However, in addition to the fact that the induction of pro- or anti-apoptotic pathways is multi-factorially regulated, the experimental conditions, such as differences in lipoprotein structures, types of cells used and culture conditions likely play a role [38], which may lead to conflicting results in different studies. The used AaPAL preparation was highly pure, the only contaminants being low amounts of LPS and BSA [28]; thus, we trusted that the observed effects were caused by AaPAL.

The levels of MCP-1, the only chemokine tested, were higher than those of TNF-α. The difference increased over time, because MCP-1 and TNF-α were positively and negatively correlated with stimulation time, respectively. However, when absolute cytokine concentrations were compared, stimulation of macrophages with increasing concentrations of AaPAL decreased the production of MCP-1. This likely depended on the declining metabolic activity of macrophages along with increasing concentrations of AaPAL, because when the MCP-1 concentrations were related to the metabolic activity, the tendency for the MCP-1 decrease was abolished. We found no previous studies on the production of MCP-1, a monocyte-attracting chemokine, by PAL-stimulated macrophages. However, *Hemophilus influenzae* PAL (P6) stimulation of human macrophages induced high levels of the neutrophil-attracting chemokine IL-8 [39]. Moreover, *E. coli* PAL upregulates the MCP-1 expression of mouse lung cells *in vivo* [36].

In our study design, AaPAL-stimulated mouse macrophages produced less IL-1β than either IL-6 or TNF-a. Similar results were found in a study where human macrophages were stimulated by purified PAL (P6) of *H. influenzae*, a phylogenetically related species of *A. actinomycetemcomitans* [39]. The incubation times were the same in both studies, but they used 0.1 µg/mL of P6, while we used 0.5–2.0 µg/mL of AaPAL. However, even at doses of 10 µg/mL, they still only detected a weak induction of IL-1β production. The design of the experimental set-up is most likely critical when studying IL-1β excretion *in vitro*, because two stimuli are needed for macrophages to release mature IL-1β [40]. Binding of Pam_3_CysSerLys_4_ to TLR2 induces only the synthesis of pro-IL-1β, which is cleaved to active cytokine by active caspase-1 [41]. However, a second stimulus is needed for the activation of caspase-1 by a cytosolic protein complex, called the inflammasome. Indeed, this two-stimulus activation model is derived from *in-* *vitro* experiments where cultured macrophages were able to activate the inflammasome and therefore produce mature IL-1β, only when treated with ATP in addition to pathogen-associated molecular patterns (PAMPs) [41]. Thus, it could be speculated that ATP treatment might have elicited more pronounced IL-1β production from AaPAL-stimulated mouse macrophages in our study.

When exposed to AaPAL, the mouse macrophages produced TNF-α, an important cytokine in systemic inflammation, more efficiently than IL-6, a pro-inflammatory cytokine which has anti-inflammatory properties in some circumstances. This was seen for each tested AaPAL concentration (0.5, 1, and 2 µg/mL) at each time point (6, 24, and 48 h), although the production of IL-6 increased linearly with time with low (0.5 and 1.0 µg/mL) AaPAL concentrations, and the peak value for TNF-α production decreased toward 48 h. Higher TNF-α production compared to IL-6 has also been reported from the human macrophage cell line THP-1 stimulated with *B. abortus* PAL [12] and *H. influenzae* PAL-stimulated human macrophages [39]. Yet, stimulation of mouse macrophages with *E. coli* PAL induces comparable levels of TNF-a and IL-6 production [22]. The pro-inflammatory effects of IL-6 are strongly related to the acute innate response, where TNF-α plays an important role; whereas, the changing micromilieu in inflammation also changes the function of IL-6 to more anti-inflammatory (for review, see ref [42].).

Our results confirmed that, similar to other bacterial lipoproteins tested *in vitro* [10,43,44], AaPAL-induced pro-inflammatory cytokine production by mouse macrophages is mediated by TLR2. The PAL of *E. coli* has also been shown to induce IL-6 and TNF-α production by mouse macrophages via TLR2 *in vivo*, as demonstrated by using a TLR2 knockout mouse model [22]. The TLR2 dependency is not restricted to macrophages because TLR2 also mediates the response of mouse lung cells to PAL, because TLR2 knockout mice are unable to upregulate the leukocyte adhesion molecules E-selectin and P-selectin as well as MCP-1 when exposed to *E. coli* PAL *in vivo* [36]. The results indicating that the synthetic lipopeptide Pam_3_CSK_4_, a well-known TLR2 agonist, did not induce the production of IL-6, TNF-α and MCP-1 through TLR2 were puzzling. The observed inability of anti-TLR2 antibody to block this specific interaction could be due to the small size of the TLR2 agonist. The anti-TLR2 antibody might not block the binding site entirely making the diffusion and binding of Pam_3_CSK_4_ to TLR2 possible.

The TLR2 dependency confirmed that the observed effects of the AaPAL purification product were due to AaPAL and not to impurities such as LPS which is recognized by TLR4. This is an important observation as both our earlier results [28] and the data published by others [22] indicate that PAL interacts strongly with LPS, leading to co-purification of these two molecules in PAL preparations. The strong interaction between LPS and AaPAL can also be observed in *A. actinomycetemcomitans* LPS preparations, in which small amounts of AaPAL can be detected (unpublished data). Thus, in *in-* *vitro* experiments other bacterial components, such as PAL, have potential to stimulate the immune cells when the inflammatory potential of purified LPS is studied [22].

Bacterial lipoproteins are established bacterial virulence factors [11–13,24,26,36] that function as inflammatory stimulants in infection and disease progression. Our study is the first to show pro-inflammatory effects of AaPAL, an outer membrane lipoprotein of a major periodontal pathogen, *A. actinomycetemcomitans*. As numerous Gram-negative species are associated with periodontitis, it is likely that they also provide PALs with respective properties for local as well as systemic inflammatory stimulation. AaPAL has the potential to activate macrophages to produce cytokines, thus initiating the innate immune response *in vivo*. The apoptotic activity of AaPAL against macrophages implies a possible role in atheroma development as macrophage apoptosis is a common event in all stages of atherosclerosis [45]. Particularly, in the late lesion when the phagocytosis of apoptotic macrophages is impaired, the inflammation and instability of the plaque is increased [45]. We previously demonstrated through an *ex-* *vivo* study design that living *A. actinomycetemcomitans* cells release free-soluble AaPAL [14,27]. Similar to other bacterial lipoproteins [46,47], it can be expected that this small molecule can cross blood vessel barriers while the bacteria remain growing locally. As most data of the systemic effects of bacterial components in periodontitis are derived from studies on LPS, our results convey knowledge of the pro-inflammatory capability of another conserved outer membrane component in periodontal species. Although results from *in* *-vitro* studies must be cautiously related to complex *in-* *vivo* processes, our study may shed some light on the still unclear mechanisms of how a local low-grade infection could contribute to the development of systemic disease, such as cardiovascular disease.
